# An integrated approach using orthogonal analytical techniques to characterize heparan sulfate structure

**DOI:** 10.1007/s10719-016-9734-7

**Published:** 2016-10-22

**Authors:** Daniela Beccati, Miroslaw Lech, Jennifer Ozug, Nur Sibel Gunay, Jing Wang, Elaine Y. Sun, Joël R Pradines, Victor Farutin, Zachary Shriver, Ganesh V. Kaundinya, Ishan Capila

**Affiliations:** Momenta Pharmaceuticals Inc., 675 West Kendall Street, Cambridge, MA 02142 USA

**Keywords:** Heparan sulfate, Domains, Structure, Analytical, NMR, MS, HPLC

## Abstract

Heparan sulfate (HS), a glycosaminoglycan present on the surface of cells, has been postulated to have important roles in driving both normal and pathological physiologies. The chemical structure and sulfation pattern (domain structure) of HS is believed to determine its biological function, to vary across tissue types, and to be modified in the context of disease. Characterization of HS requires isolation and purification of cell surface HS as a complex mixture. This process may introduce additional chemical modification of the native residues. In this study, we describe an approach towards thorough characterization of bovine kidney heparan sulfate (BKHS) that utilizes a variety of orthogonal analytical techniques (*e.g.* NMR, IP-RPHPLC, LC-MS). These techniques are applied to characterize this mixture at various levels including composition, fragment level, and overall chain properties. The combination of these techniques in many instances provides orthogonal views into the fine structure of HS, and in other instances provides overlapping / confirmatory information from different perspectives. Specifically, this approach enables quantitative determination of natural and modified saccharide residues in the HS chains, and identifies unusual structures. Analysis of partially digested HS chains allows for a better understanding of the domain structures within this mixture, and yields specific insights into the non-reducing end and reducing end structures of the chains. This approach outlines a useful framework that can be applied to elucidate HS structure and thereby provides means to advance understanding of its biological role and potential involvement in disease progression. In addition, the techniques described here can be applied to characterization of heparin from different sources.

**Electronic supplementary material:**

The online version of this article (doi:10.1007/s10719-016-9734-7) contains supplementary material, which is available to authorized users.

## Introduction

Heparan sulfate (HS) is a member of the glycosaminoglycan family, ubiquitously found at the surface of mammalian cells, where it plays a role in mediating the cell’s interaction with its extracellular environment. HS is generally present as a proteoglycan (HSPG), *i.e.* covalently attached to a core protein, and it is involved in different biological processes, including cell-cell communication, binding of lipoprotein lipases and lipoproteins to cell surfaces, regulation of growth factors and cytokines, and binding to antithrombin to support blood flow across the vascular endothelium [1–5]. HS also has a regulatory role in pathological conditions, such as inflammation, tumor onset, progression, and metastasis [6, 7].

Heparan sulfate is a polydisperse mixture of linear polysaccharides consisting of repeating units of 1 → 4-linked glucosamine and uronic acids that can be variably sulfated during biosynthesis. Heparan sulfate biosynthesis is a complex process that starts with the action of D-glucuronyl and *N*-acetyl-D-glucosaminyl transferase to generate a copolymer of alternating H_NAc_ and G, which subsequently undergoes modifications by at least four families of sulfotransferases and one epimerase. Enzyme specificity during HS biosynthesis dictates the positioning of sulfate groups; also, the expression levels of different isoforms of HS biosynthetic enzymes contribute to the synthesis of specific saccharide sequences characteristic of each cell type [8].

Despite the significant potential for diversity in HS, the concerted action of the biosynthetic machinery seemingly generates putative block structures [9, 10]. In this model, HS is thought to be composed of NA domains, which are dominated by repeating units of alternating G and H_NAc_, and NS domains, constituted of I_2S_ and H_NS_ residues. Mixed or transition domains that alternate sulfated and non-sulfated monosaccharides separate the NA domains from the NS domains [11, 12].

Due to their higher charge density, the NS and transition domains constitute the preferred protein-binding regions of HS [13–15]. The major role of the NA domains is to confer structural flexibility to the HS backbone and facilitate interaction of more sulfated regions of HS with proteins [16]. NA domains are believed to lack specific binding properties, although heparanases seem to use differences in sulfate content between NS and NA domains to orient themselves and reach the sites of cleavage [17].

In addition, specific HS structures and sequences have been identified as critical for interaction with antithrombin, selectin, FGF-1 and FGF-2 [18, 19].

Many HS-protein interactions are believed to depend on the overall organization and spacing of NS domains, which regulate oligomerization or formation of ternary complexes [20, 21]. So far, little is known about the distribution of NS domains along or at the non-reducing end of the HS chains, while several studies indicate that NA sequences are located close to the protein-linkage region [22, 23].

A detailed understanding of the structure-function relationship for HS would require elucidation of fine structure, overall composition, spacing, and distribution of NS, NA, and transition domains along the HS chain. In this paper, multiple analytical tools are applied to provide a comprehensive understanding of bovine kidney HS (BKHS) structure. First, the overall disaccharide composition of BKHS is examined and quantified using orthogonal analytical methods (2D NMR on intact chains, and enzymatic digestion followed by IP-RPHPLC and LC-MS). Identification of minor residues such as epoxide, galacturonic acid, non-sulfated glucosamine, and glucuronic acid at the non-reducing end of the HS chain is described in detail.

Next, BKHS is separated into chains of different length to identify a potential relationship between degrees of polymerization and distribution of NA, NS, and transition domains. Finally, BKHS is digested with *heparinases* (*Hep*) I or III, and the digested mixture is analyzed by gel permeation chromatography-mass spectrometry (GPC-MS) to allow characterization of NA, NS, and transition domains both in terms of chain length and finer structure. The results of GPC-MS, LC-MS and NMR techniques are used to perform structural analysis of non-reducing ends and reducing ends of HS chains, and to identify unusual structures, such as phosphorylated residues within the linkage region.

This analysis builds on previous studies that have demonstrated sequencing of “simple” glycosaminoglycans, such as bikunin [24], and provides a systematic analysis of the more structurally complex HS. Given the complexity of HS, employment of multiple, orthogonal analytical approaches to obtain distinct levels of information on the polysaccharide chain provides important structural insights in a more comprehensive manner than is possible from a single analytical approach. Therefore, this approach outlines a useful framework that can be applied to elucidate structural properties of a heparan sulfate mixture.

## Materials and methods

### Enzymatic digestion of BKHS samples for IP-RPHPLC and LC-MS analysis

BKHS (1 mg, Seikagaku) was digested at 30 °C for 16 h using a cocktail of *Bacteroides* *Heparinases* I, III, and IV manufactured in house. The digested sample was then incubated for 6 h at 30 °C simultaneously with 2-*O-*sulfatase and Δ^4,5^ -glycuronidase. On completion of digestion, the sample was frozen and lyophilized. *Flavobacterium* *Heparinases* (I, II and III) may also be used instead of the cocktail of *Bacteroides* *Heparinases* I, III, and IV [25].

### Ion-pairing reverse phase HPLC analyses

Digested BKHS samples were analyzed by IP-RPHPLC using 30 mM tetra-*n*-butyl ammonium chloride (TBA) as the ion-pairing reagent in 15 % acetonitrile (ACN). The digested samples were separated using an analytical C18 Discovery column (4.6 × 250 mm, Supelco) maintained at 25 °C, with a flow rate of 0.7 mL/min over 130 min of total run time, with a gradient ranging from 0 to 1 M NaCl. The elution profile was monitored by UV absorption at 232 nm.

### LC-MS analysis

Chromatographic separation was performed on a 0.3 mm × 250 mm C18 polymeric silica column (thermostated at 17 °C) at a flow rate of 4 μL/min using a Ultimate 3000 capillary HPLC system (Dionex). The mobile phase consisted of (A) 8 mM dibutylammonium acetate in 100 % water, (B) 8 mM dibutylammonium acetate in 70 % methanol/30 % water and (C) 90 % acetonitrile/10 % water. Samples were eluted with the following step gradient: 0 % B for 4 min, 9 % B for 20 min, 20 % B for 21 min, 32 % B for 19 min, 48 % B for 17 min, 63 % B for 14 min, 100 % C for 14 min, and 100 % A for 20 min. To improve ionization, post-column addition of aqueous methanol was carried out using a secondary binary solvent system. Mass spectra were acquired in negative mode with a quadrupole time-of-flight mass spectrometer (Waters-Micromass, UK). Data acquisition was controlled by Masslynx 4.1, according to the following settings: source temperature 100 °C, desolvation temperature 200 °C, capillary voltage 2.4 V, cone voltage 15 V and collision energy 1.7 V.

### Size fractionation

10 mg of BKHS were fractionated on a Superdex 75 column (100 mL column volume) and eluted with 5 mM sodium phosphate dibasic and 150 mM sodium chloride, pH 7.0, at a linear flow rate of 15 cm/h. Fractions were buffer-exchanged with 10 % ethanol using Sephadex G-10 and lyophilized to a dry powder.

### NMR analysis

The mono- and two-dimensional spectra of BKHS were recorded at 298 K, with a 600 MHz Bruker Avance spectrometer equipped with a 5-mm triple-resonance inverse cryoprobe. Before spectra acquisition, HS was dissolved at 2 mg/150 μL of D_2_O (99.9 %) and sonicated for 30 s to remove air bubbles.

^1^H–NMR spectrum was acquired with presaturation of the residual water signal, with a recycle delay of 5 s, for 64 scans. COSY spectrum was recorded with presaturation of the water signal, for 8 scans of 320 increments. TOCSY spectrum was acquired in phase sensitive mode with 80 ms of DIPSI-2 mixing, for 24 scans of 320 increments. For COSY and TOCSY spectra, the matrix size was zero filled to 2 K × 2 K prior to Fourier transformation. HSQC spectrum was recorded with sensitivity enhancement and carbon decoupling during acquisition, for 64 scans of 256 increments. The polarization transfer delay was set with a ^1^ *J*_C-H_ coupling value of 155 Hz. For HSQC spectra, the matrix was zero filled to 2 K × 1 K prior to Fourier transformation.

### GPC-MS analysis

BKHS (0.6 mg) was digested at 30 °C for 16 h with *Bacteroides Heparinases* I (5 IU/mg) or III (2 IU/mg). On completion of digestion, the sample was frozen, lyophilized, and separated on a chromatographic system consisting of the Ultimate 3000 with Dual-Ternary Capillary HPLC pumps (Dionex) and two 4.6 mm × 300 mm, 4 μm gel permeation TOSOH TSKgel SuperSW2000 columns placed in series and equilibrated at 25 °C. 100 mM ammonium acetate was used as eluent at the constant flow-rate of 100 μl/min delivered by ternary solvent system. The wavelength of the UV detector (Dionex) was set at 232 nm, followed by the Dionex 2 mm Anion Self-Regenerating Suppressor 300 (ASRS-300 (2-mm)) using water as regenerant. Electrolysis of water in the ASRS-300 was driven by the Dionex SRS controller using the 100 mA current. The flow was post-column controlled by QuickSplit adjustable flow splitters (Analytical Scientific Instrument) to a constant splitting ratio of 1:10.

MS analysis was carried out on a quadrapole time-of-flight (Q-TOF) mass spectrometer (Waters) equipped with an electrospray ionization source (ESI). Mass spectra were acquired in negative “W″ ion mode; N_2_ was used as a desolvation gas as well as a nebulizer. Conditions for ESI-MS were as follows: source temperature 100 °C, desolvation temperature 300 °C, capillary voltage 2.4 kV, desolvation gas flow 250 L/h, cone voltage 7 V, cone gas flow 100 L/lr, and collision energy set to 1.2 V. MS spectra were generated by scanning the range of m/z 70–1400, scan time 1.0 s, interscan time 0.1 s. and pusher cycle time 99 μs.

### Digestion with alkaline phosphatase

HS (200 μg), previously digested with *Bacteroides Heparinase* III, was diluted in 1 mL of 50 mM Tris, pH 8.8, containing 1 mM MgCl_2_. Six units of alkaline phosphatase (from *E coli*, Sigma-Aldrich) were added and the solution was incubated at 37 °C overnight.

## Results

### Composition analysis by IP-RPHPLC, NMR, and LC-MS

In this study, ion-pairing reverse phase high performance liquid chromatography (IP-RPHPLC) was used to determine disaccharide composition of BKHS after exhaustive digestion with a cocktail of *heparinases*, as reported previously. Peaks were identified by co-injection with commercial standards, or through peak isolation and characterization by mass spectrometry (MS) and nuclear magnetic resonance spectroscopy (NMR). The disaccharide composition of BKHS is reported in Table 1. Consistent with characterization of HS from other sources [26], IP-RPHPLC data indicate that six disaccharides constitute more than 90 % of BKHS (based on area percentage): ΔUH_NAc_, ΔUH_NS_, ΔUH_NAc6S_, ΔUH_NS6S_, ΔU_2S_H_NS_, and ΔU_2S_H_NS6S_ where ΔU indicates the formation of a Δ^4,5^ glycuronidate as a result of enzymatic cleavage. A tetrasaccharide corresponding to the anti-thrombin binding site, *i.e.* ΔUH_NAc6S_GH_NS3S6S_ [27], was also identified. As previously demonstrated, this structure is resistant to further enzymatic cleavage due to the presence of a reducing glucosamine carrying a sulfate group at the 3-*O* position [28].

**Table 1:** Disaccharide composition of BKHS determined by IP-RPHPLC

	Relative Area (%)
∆UH_NAc_	52.3
∆U_gal_H_NS_	0.5
∆UH_NS_(α)	14.7
∆UH_NS_(β)	1.8
∆UH_NAc6S_	10.4
∆U_2S_H_NAc_ (α)	0.3
∆U_2S_H_NAc_ (β)	0.5
∆UH_NS3S_	0.1
∆UH_NS6S_ (α)	5.4
∆U_gal_H_NS6S_ (α)	0.1
∆UH_NS6S_ (β)	0.6
∆U_2S_H_NS_ (α)	6.9
∆U_2S_H_NS_ (β)	0.9
∆U_2S_H_NAc6S_(α)	0.1
∆U_2S_H_NAc6S_(β)	0.1
∆UHN_Ac6S_GH_NS,3S_	0.5
∆U_2S_H_NS6S_ (α)	4.4
∆UH_NAc6S_GH_NS3S6S_ (α)	0.1
∆U_2S_H_NS6S_ (β)	0.4

**Fig. 1:**
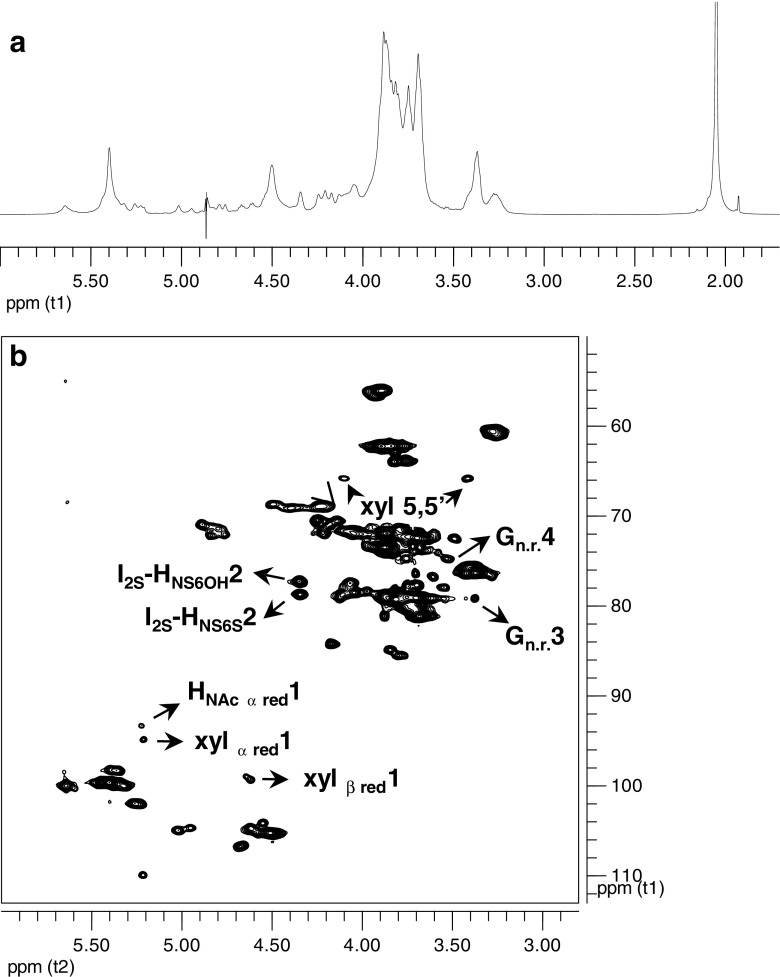
**a** ^1^H–NMR and **b** HSQC spectra of BKHS. Signals due to the reducing end (xyl α/β and H_NAc_ α), non-reducing end (G _n.r._), and differently substituted internal I_2S_ are indicated

To complement and extend results obtained by IP-RPHPLC, intact BKHS was analyzed by mono- and di-dimensional NMR. ^1^H–NMR and ^1^H-^13^C heteronuclear single quantum coherence (HSQC) spectra of BKHS are reported in Fig. 1a, b, respectively. Assignment of HSQC cross peaks was based on literature data [29, 30] and analysis of COSY, TOCSY, and ROESY spectra (data not shown). Taken together, the NMR data identify H_NS_-(I_2S_/I) and H_NS/NAc_-(G) as the major glucosamine components of BKHS, and H_NS3S_, H_NH2_, and (H_NAcαred_) as minor glucosamine components (Table 2). The most prevalent uronic acids include I_2S_-(H_NS_), I-(H_NS/NAc_), and G-(H_NS/NAc_). Although most of these residues are identified by the chemical shifts of H1/C1, the position of the H2/C2 peaks allows discrimination between I_2S_-(H_NS6OH_) and I_2S_-(H_NS6S_) (4.349/77.32 and 4.341/78.72 ppm, respectively) [31]. The presence of small amount of epoxide is indicated by a weak signal at 5.41/97.1 ppm. Epoxide is an unnatural residue, generated by the basic reagents used during isolation of HS from the protein core. Treatment with bases is known to cause 2-*O*-desulfation of uronic acids with concomitant formation of epoxide [32].

**Table 2:** Relative percentage of variously substituted glucosamine and uronic acid in BKHS determined by HSQC analysis

Monosaccharides^1^	Composition (mole %)
Glucosamine^2^	
H_NS_-(I_2S_)	11.5
H_NS_-(I)	9.6
H_NS_-(G)	10.6
H_NAc_-(G)	64.8
H_NS3S_	0.6
H_NH2_	1.5
H_NAc_ αredox	1.4
H_NX6OH_^3^	75.0
H_NX6S_^3^	25.0
Linkage region^2^	
LR	4.7
Uronates^4^	
I_2S_-(H_NS6OH_)	10.1
I_2S_-(H_NS6S_)	7.7
I-(H_NX6S_)^3^	7.4
I-(H_NX6OH_)^3^	4.3
G-(H_NS_)^5^	12.8
G-(H_NAc_)^5^	57.0
G-(H_NS3S_)^5^	n.d.
Epoxide	0.7
GalA	n.d.

^1^Residues reported in brackets indicate the monosaccharide downstream of the quantified glucosamine or uronic acid residue

^2^Mole % of the total glucosamine content

^3^X = S or Ac

^4^Mole % of the total uronic acid content

^5^ The amount of G at the non-reducing end is calculated as 6.4 % of the total uronic acid content

Besides mono/disaccharide composition, NMR analysis provides useful information regarding the non-reducing and reducing end of BKHS chains. Glucuronic acid located at the non-reducing end is differentiated from internal glucuronic acid by the distinct chemical shift of its H3/C3 and H4/C4 signals. While peaks corresponding to internal glucuronic acid resonate at 3.73/79.2 and 3.83/79.5 ppm in a spectral region crowded with signals, non-reducing glucuronic acid exhibits clear resonances at 3.55/77.9 (H4/C4) and 3.53/74.7 ppm (H3/C3) [33].

Signals of monosaccharides belonging to the linkage region are observed at 4.67/106.8 ppm (GlcA-*β*1–3- and Gal-*β*1–3-) and 4.55/104.1 ppm (Gal-*β*1–4-). The presence of a xylose residue is indicated by H5,5′/C5 cross-peaks at 3.42/65.8 and 4.10/65.8 ppm. Signals at 5.22/94.8 ppm and 4.62/99.3 ppm are assigned to the H1/C1 of the *α* and *β* form of xylose, respectively, which is present as a reducing sugar. This observation suggests that the conditions commercially used to isolate BKHS release the saccharide chain from the Ser residue of the proteoglycan core. Additional signals at 5.23/93.4 ppm and 6.37/98.9 ppm are attributed to reducing *N*-acetyl glucosamine (*α* and *β*, respectively), providing evidence of the presence of BKHS chains lacking the linkage region sequence. Quantitative composition analysis is performed by integrating the relevant peaks in the HSQC spectrum of BKHS (Table 2). The amount of linkage region and *N*-acetyl glucosamine at the reducing position is used to estimate an average chain length of 16 disaccharides. The relative amount of glucuronic acid at the non-reducing end of the chains is comparable to the amount of reducing end residues (*i.e.*, linkage region plus H_NAc_ redox), indicating that in commercial BKHS the majority of the chains start with a G residue.

The monosaccharide composition determined either by IP-RPHPLC and 2D NMR is compared in Supplementary Table 1. Although IP-RPHPLC analysis has the advantage of requiring less material compared to NMR, the latter provides additional structural information, including epimerization at the C5 position of the uronic acid within different disaccharide moieties. In addition, unlike IP-RPHPLC, 2D NMR detects residues lacking UV absorbance, *e.g.*, glucuronic acid present at the non-reducing end of chains. Finally, NMR is better suited to characterize the linkage region and to determine the presence of residual serine, oxidized serine, or the lack of it. Identification by HPLC is in this case hampered by the lack of suitable standards and by the fact that the current HPLC conditions are optimized to separate sulfated disaccharides and, consequently, are not efficient in isolating neutral residues like the linkage region. This is also the reason why, although NMR shows presence of small amounts of unsubstituted glucosamine (H_NH2_), such a residue could not be detected by IP-RPHPLC (the standard ∆UH_NH2_ elutes in the void volume).

Analysis of IP-RPHPLC and NMR data indicates general agreement between the two techniques regarding sulfation level and overall composition. However, IP-RPHPLC analysis shows presence of galacturonic acid (ΔU_gal_), while NMR detects 2,3-epoxide [34]. The root cause for this apparent discrepancy could be attributed to the use of heparin lyase enzymes prior to HPLC analysis, which may result initially in hydrolysis of the epoxide to galacturonic acid, then in conversion of galacturonic acid to ΔU_gal_ through H5 abstraction and glycosidic bond cleavage. Preliminary experiments performed with various enzyme cocktails seem to confirm this hypothesis (data not shown).

To detect structures present in trace amounts, and complete compositional analysis of BKHS, a LC-MS method based on ion-pairing reversed phase high performance liquid chromatography (IP-RPHPLC) coupled to electrospray ionization time-of-flight mass spectrometry (ESI-TOF-MS) was applied to BKHS digested with heparin lyases. This analysis confirmed the major constituents previously detected by IP-RPHPLC (see Supplementary Table 2). Furthermore, the higher degree of sensitivity of ESI-MS enabled identification of additional species below the detection limit of IP-RPHPLC, *e.g.*, unsaturated tetrasaccharides with different levels of sulfation and acetylation. These tetrasaccharides contain a 3-*O*-sulfated glucosamine or an epoxide moiety, which explains their resistance to complete enzymatic digestion. Although epoxide residues may be converted into galacturonic acid by *heparinases*, trace amounts can be detected by ESI-MS. Importantly, through the detection of disaccharides that do not contain a Δ^4,5^ glycuronate, LC-MS analysis provides information regarding the non-reducing end of chains. Two types of non-reducing end structures are identified: saturated trisaccharides starting with a glucosamine residue and saturated di/tetrasaccharides starting with an uronic acid moiety. These saccharides present different levels of acetylation and sulfation, ranging from zero to three sulfates per disaccharide and from two to five sulfates per trisaccharide. A fragment present at the reducing end of the chain is also identified, *i.e.* ΔU-Gal-Gal-Xyl-OH. A molecular mass of 337.10 Da assigned to the building block ∆UH_NH2_ provides evidence of the presence of unsubstituted glucosamine, as previously shown by 2D NMR data.

### Size fractionation

To determine whether disaccharide composition changes as a function of chain length, BKHS was separated by SEC into seven fractions with different elution times. These fractions were then digested with a heparinase cocktail and analyzed by IP-RPHPLC (Table 3). Disaccharide composition data indicate that shorter chains contain a higher amount of residues belonging to the NA region (*i.e.* enriched in ΔUH_NAc_) as well as residues attributed to the NS region (*i.e.* ΔU_2S_H_NS6S_ and ΔU_2S_H_NS_). Shorter chains contain fewer residues of the transition regions, as indicated by lower levels of ΔUH_NS_, ΔUH_NS6S_, and ΔUH_NAc6S_. Comparison of *N*-sulfation, 6-*O*-sulfation, and 2-*O*-sulfation levels among chains of different length indicates that shorter chains contain less 6-*O* and *N*-sulfates than longer chains, but are more sulfated at the 2-*O* position (Table 4).

**Table 3:** IP-RPHPLC analysis of BKHS and its fractions (Rel. Area %). Fractions are reported in order of elution: Pool 1 + 2 corresponds to the fraction with the highest MW, Pool 10 to the fraction with the lowest MW. The MW progressively decreases form Pool 1 + 2 to Pool 10

	Chain Region	BKHS	Pool 1 + 2	Pool 3	Pool 4	Pool 5	Pool 7	Pool 8	Pool 10
ΔUH_NAc_	NA domain	55.2	52.0	53.5	53.5	54.3	56.4	58.1	58.1
ΔUH_NS_	Transition	17.7	20.1	19.5	19.0	18.3	17.3	16.4	15.9
ΔUH_NAc6S_^1^	Transition	10.3	12.0	12.0	11.0	10.6	9.2	8.9	7.9
ΔUH_NS6S_	Transition	6.1	6.9	6.0	6.3	6.3	6.1	5.6	6.0
ΔU_2S_H_NS_	NS domain	6.1	4.6	4.8	5.6	5.9	6.2	6.4	6.9
ΔU_2S_H_NS6S_	NS domain	4.6	4.4	4.2	4.5	4.5	4.8	4.6	5.2

^1^Glucosamine 6-*O*-sulfotransferase sulfates H_NAc_ residues provided that there is a H_NS_ residue on either the upstream or the downstream disaccharides [35]. According to the enzyme specificity, we can assume that all the ΔUH_NAc6S_ residues are in the transition region or just at the border of it

^2^ The relative standard deviation (RSD) for the peaks measured by this method is ≤1 %, which basically corresponds to a change in peak area of ±0.6 for the most abundant peak and about ±0.1 or less for the lower abundance peaks. Therefore, the changes observed across fractions are larger than what would be expected by method variability

**Table 4:** Relative percentage of *N*-sulfation vs *N*-acetylation, 6-*O*-sulfation, and 2-*O*-sulfation in BKHS and its fractions (IP-RPHPLC data). Fractions are reported in order of elution: the MW progressively decreases form Pool 1 + 2 to Pool 10

	Intact	Pool 1+ 2	Pool 3	Pool 4	Pool 5	Pool 7	Pool 8	Pool 10
*N*-Sulfation	34	37	35	36	36	34	33	33
*N*-Acetylation	66	64	66	64	65	66	67	66
6-*O*-Sulfation	21	24	22	22	22	20	19	19
2-*O*-Sulfation	11	9	9	10	10	11	11	12

### GPC and GPC-MS analysis of HS digested with HepI or HepIII

To examine chain composition in finer detail, BKHS was initially digested with either *Hep* I, an enzyme that cleaves highly sulfated regions, or *Hep* III, which cleaves *N*-acetylated and undersulfated domains [36, 37]. After overnight digestion, reaction products were separated by GPC and the UV elution profile at 232 nm was integrated to determine the fragments composition and length distribution (Supplementary Figs. 1 and 2, Supplementary Tables 3 and 4). Digestion of BKHS with *Hep* I degrades NS domains into disaccharides while substantially preserving the NA and the transition domains of the BKHS chains. GPC of *Hep* I-digested BKHS indicates that fragments containing 20 or more monosaccharides represent a significant portion of this sample. Conversely, digestion of BKHS with *Hep* III provides mainly disaccharides; tetra- to dodeca-saccharides constitute in this case a small portion of the total mixture.

Fragments generated by *Hep* I and *Hep* III digestion were consequently analyzed by GPC-MS (Supplementary Tables 5 and 6, respectively). The relative abundance of each fragment was derived from peak intensities determined by applying the MaxEnt3 (Maximum Entropy) algorithm and after application of correction factors to selected residues (see Supplementary Information**)**. Correction factors are introduced to compensate for variations in ionization efficiency of saccharides containing different molar quantities of sulfate groups. Experimentally, using isolated saccharide standards, it was established that differences in ionization due to sulfation are more significant for shorter fragments (*i.e.,* disaccharides). Given this result, it was considered more robust to base disaccharide quantification on UV absorbance; therefore, the disaccharide ratio determined by MS was adjusted to reflect abundances determined by UV. The same correction factors were applied to both saturated and unsaturated disaccharides.

Fragments listed in Supplementary Tables 5 and 6 can be traced back to their position within the BKHS chains: saturated residues derive from the non-reducing end of the chain; residues with a Δ^4,5^ moiety are originally internal to the chain; residues of formula ΔU-Gal-Gal-Xyl-OH belong to the linkage region at the reducing end of the chain. Monosaccharides and residues of formula H-(U-H)_n_ are believed to derive from the non-reducing end of the chain. As will be described below, digestion of HS followed by GPC-MS analysis is a useful tool to provide information regarding the NS, NA, and transition domains internal to the chain, as well as the non-reducing and reducing ends of the chain.

### Characterization of transition and NA domains

Digestion with *Hep* I largely preserves NA domains and transition domains, while it hydrolyzes NS domains to component disaccharides. The GPC profile of BKHS digested with *Hep* I indicates that fragments with a degree of polymerization (Dp) from 8 to 20 are approximately equally represented, and chains longer than Dp20 constitute about 20 % of the entire mixture. Because NA domains are defined as repeating units of G-HNAc residues, fragments generated by *Hep* I can be undoubtedly classified as belonging to NA if they contain a number of acetyl groups equal to (Dp-N)/2 and at least two sulfated residues, which supposedly reside at the border of the NA region and constitute the site of action of *Hep* I. Residues that are assigned to NA domains are marked with an asterisk in Supplementary Table 5. The presence of fragments with a Dp ≥ 20 indicates that BKHS contains NA domains longer than 18 saccharides. Fragments that possess a number of acetyl groups ≤ (Dp-N)/2 are assigned to the transition regions. Transition regions are characterized by significantly higher 2-*O* sulfation and/or 6-*O* sulfation than NA domains, as demonstrated by the presence of structures of formula Dp20,Ac7,S8,Δ and Dp18,Ac7,S6,Δ. Interestingly, the transition domains appear to be of comparable length to the NA domains.

### Characterization of NS domains

By preserving sulfated regions, digestion of BKHS with *Hep* III allows characterization of NS domains (Supplementary Table 6). The maximum length of fragments obtained after digestion with *Hep* III is Dp12, providing evidence that within BKHS the NS domains are significantly shorter than the NA or the transition domains. The number of sulfate groups per fragment of the same length indicates that 6-*O*-sulfation is highly variable: some fragments are completely 6-*O*-sulfated (*e.g.,* Dp8,10S,0Ac,Δ) whereas others are 6-*O-*desulfated (*e.g.,* Dp8,7S,0Ac,Δ). Interestingly, shorter fragments (Dp ≤8) are more likely to present acetyl groups at the border of the NS domains.

### Characterization of non-reducing end

Characterization of BKHS non-reducing ends is performed by GPC-MS analysis of saturated residues obtained after digestion of BKHS with *Hep* I and *Hep* III (Supplementary Tables 5 and 6, respectively). It was previously reported that non-reducing ends of HS chains are mainly sulfated [38]. The GPC-MS and HSQC data reported here indicate that they also contain *N*-acetylglucosamine in significant amounts, and that the most abundant residue observed at the non-reducing end of BKHS chains is glucuronic acid (G). Despite the presence of *N*-acetylglucosamine, non-reducing ends do not contain NA domains, but NS sequences ranging from 8-mer to 12-mer, or transition domains up to 8-mer. Although complete structural elucidation goes beyond the MS capabilities of this experimental set-up, assignment of the predominant non-reducing end structures is proposed by combining information derived from GPC-MS, NMR, the known substrate specificities of *Hep* I and *Hep* III [36, 37], and observations from previous studies on the biosynthetic machinery of HS. Given this set of information, assignment of the non-reducing end structures is proposed in Tables 5 and 6.

**Table 5:** GPC-MS Analysis of non-reducing end residues of BKHS digested by *Hep* I

Composition	Relative intensity	Proposed Sequence
Dp2, Ac0, S1, saturated	1.14	G-H_NS_
Dp2, Ac0, S2, saturated	2.48	G-H_NS6S_
Dp4, Ac1, S2, saturated	0.94	G-H_NAc_-G^1^-H_NS6S_
Dp4, Ac0, S3, saturated	0.42	G-H_NS6X_–G/I-H_NS6X_
Dp4, Ac1, S3, saturated	0.38	G-H_NAc6S_–G^1^-H_NS6S_
Dp4, Ac0, S4, saturated	0.17	G-H_NS6S_–G/I-H_NS6S_
Dp6, Ac1, S3, saturated	0.13	G-H_NAc6X_^2^^,^^3^-G^1^-H_NS6X_^2^^,^^3^-G/I-H_NS6X_
Dp6, Ac1, S4, saturated	0.09	G-H_NAc6X_^2^^,^^3^-G^1^-H_NS6S_^2^^,^^3^-G/I-H_NS6S_
Dp6, Ac2, S2, saturated	0.10	G-H_NAc_-G^1^-H_NAc6X_^2^-G-H_NS6X_
Dp8, Ac2, S4, saturated	0.02	Not determined

X = S or OH

^1^The specificity of the enzymes that synthesize HS suggests that immediately downstream from HNAc there must be a G [51]. Exception have been found, but these structures should constitute a minority

^2^Glucosamine 6-*O*-sulfotransferase sulfates H_NAc_ residues provided that there is a H_NS_ residue on either the upstream or the downstream disaccharides

^3^the positions of H_NAc_ and H_NS,6X_ can be interchanged

**Table 6:** GPC-MS Analysis of non-reducing end residues of HS digested by *Hep* III

Composition	Relative intensity	Proposed Sequence
Dp2, 0S, 1Ac, saturated	0.83	G-H_NAc_
Dp2, 2S, 0Ac, saturated	0.25	G-H_NS6S_
Dp8, 6S, 2Ac, saturated	0.11	Not determined
Dp8, 7S, 0Ac, saturated	0.14	G-(H_NS_-I_2S_)_3_-H_NS_
Dp8, 8S, 0Ac, saturated	0.21	G-(H_NS6X_–I_2S_)_3_-H_NS6X_
Dp8, 9S, 0Ac, saturated	0.12	G-(H_NS6X_–I_2S_)_3_-H_NS6X_
Dp8, 10S, 0Ac, saturated	0.07	G-(H_NS6S_–I_2S_)_3_-H_NS_
Dp10, 9S, 0Ac, saturated	0.07	G-(H_NS_-I_2S_)_4_-H_NS_
Dp10, 10S, 0Ac, saturated	0.09	G-(H_NS6X_–I_2S_)_4_-H_NS6X_
Dp10, 11S, 0Ac, saturated	0.04	G-(H_NS6X_–I_2S_)_4_-H_NS6X_
Dp12, 11S, 0Ac, saturated	0.02	G-(H_NS_-I_2S_)_5_-H_NS_
Dp12, 12S, 0Ac, saturated	0.02	G-(H_NS6X_–I_2S_)_5_-H_NS6X_

X = S or OH

### Characterization of reducing end

Confirming NMR data, LC-MS and GPC-MS identify ΔU/G-Gal-Gal-Xyl-OH as the most abundant residue attributed to the linkage region (Supplementary Table 6). A residue of mass 712.14 Da, corresponding to the mass of ΔU-Gal-Gal-Xyl-OH plus 80 mass units is also observed in BKHS digested with *Hep* III. To determine whether the 80 mass units could be attributed to a phosphate (PO_3_^−^) or to a sulfate (SO_3_^−^) group, the sample was digested with alkaline phosphatase and analyzed by GPC-MS. Disappearance of the peak at *m/*z 711.14 [M-H]^−^ and concomitant increase of the peak at *m/*z 631.21 [M-H]^−^ provided evidence of the presence of a phosphorylated residue. Fragmentation analysis showed that the phosphate group is carried by xylose (data not shown). Interestingly, the presence of phosphorylated linkage region has been previously reported only in bovine lung HS [39]. Phosphorylated linkage region is present only in one of the two commercial lots of BKHS analyzed. This may indicate that the amount of phosphate is highly variable, or that the isolation procedure may not consistently preserve these structures.

No residues containing the linkage region were observed by GPC-MS analysis of BKHS digested with *Hep* I. Based on this analysis, the disaccharide sequences close to the linkage region are believed to be mainly non-sulfated, and therefore not easily degraded by *Hep* I. The linkage region may be present in long fragments not easily detectable by GPC-MS analysis [40].

## Discussion

Elucidation of GAG chain sequences is a challenging task due to the inherent variability imparted by a non-template driven biosynthetic process coupled with the difficulty of isolating individual HS chains for structural characterization. The theory that GAGs may possess a unique sequence is a subject of considerable debate. Although it is known that biosynthetic enzymes are responsible for organizing HS chains into regions of high sulfation separated by domains of low sulfation [41], it is still unclear whether they possess enough specificity to arrange such domains according to a specific pattern. Recent studies conducted on bikunin GAG indicate that this simple proteoglycan possesses a defined sequence [24]: because different members of the glycosaminoglycan family share a common biosynthetic pathway, results obtained on bikunin GAGs seem to suggest that other GAGs could have well-defined sequences.

To provide additional characterization of HS and to develop a protocol for analysis of complex polysaccharide mixtures, orthogonal analytical methods were optimized and applied to BKHS structural elucidation. The initial efforts focused on implementation of orthogonal analytical methods to determine the fine structure of BKHS, *i.e.*, its mono−/disaccharide composition. Three different techniques were evaluated: 2D NMR HSQC analysis of intact BKHS, and IP-RPHPLC and LC-MS analysis of BKHS digested with a cocktail of heparinases. By detecting major components as well as structures present in small amount such as H_NH2_, H_NS3S_, ∆U_2S_H_NAc6X_, or ∆UH_NAc6S_GH_NS3S6X_, HSQC and IP-RPHLC provide a snapshot of the structural microheterogeneity of BKHS. These techniques also identify unnatural moieties generated by the chemical treatment used to release HS from the proteoglycan core, *i.e*. 2,3-epoxide and GalA, allowing control over the extent of modification caused by the isolation process [32]. HSQC and IP-RPHLC methods can easily be applied to determine sulfation levels at specific positions (*i.e*., at the 2*-O* position of uronic acids, or at the *N*- and 6-*O* position of glucosamine residues), making them suitable techniques to compare HS from different sources or pathologic states [42, 43]. In addition, detailed monosaccharide analysis could provide insight into the post-biosynthetic fate of BKHS: the presence of significant amount of glucuronic acid at the non-reducing end of the chains rules out extensive digestion by *β*-D-glucuronidase or heparanase, enzymes that would generate chains starting with a glucosamine residue.

LC-MS analysis of HS chains digested with a cocktail of heparinases is a valuable technique to detect structures that are present in trace amount, below the limit of detection for NMR and IP-RPHPLC. When applied to the study of non-reducing end structures, LC-MS identifies free glucosamine (H_NH2_) and saturated trisaccharides starting with a glucosamine residue, in addition to saturated di/tetrasaccharides starting with a uronic acid moiety. The presence of saturated residues with different grade of sulfation indicates that non-reducing ends possess a highly variable composition. A recently developed analytical approach based on LC-MS analysis of non-reducing ends of enzymatically digested GAGs isolated from cells, blood and urine is intended to be used as a diagnostic tool to detect lysosomal enzyme deficiency [44].

IP-RPHPLC analysis of BKHS fractionated according to chain length suggests that disaccharide composition, as well as domains organization, change as a function of chain polymerization. Shorter chains seem to contain higher relative amount of disaccharides belonging to the NA and NS regions, and less residues belonging to the transition regions. Previous reports indicated that 2-*O* and 6-*O*-sulfotransferase enzymes are independently regulated during HS biosynthesis [45]. Currently, a comparison of 6-*O*-sulfation, 2-*O*-sulfation, and *N*-sulfation levels among chains of different length suggests that *N*-deacetylases, *N*-sulfotranferases, and 6-*O*-sulfotransferases are more active on longer chains, while 2-*O*-sulfotranferases modify shorter chains more extensively than longer chains. It was previously acknowledged that chains of different lengths may have different composition, and that chain length may be indicative of different biological roles of HS: neuroepithelial cells secrete shorter and simpler chains when they interact with FGF-2, and longer and more complex chains when they interact with FGF-1 [46].

To determine length, distribution, and composition of different domains, BKHS is digested with either *Hep* I or *Hep* III, and the obtained mixtures are analyzed by GPC and GPC-MS. Digestion with *Hep* I, an enzyme that preserves non-sulfated sequences, provides fragments with a degree of depolymerization ranging from 2 to more than 20 saccharides, which can be assigned by MS to either NA or transition domains. Taken together, MS data, degree of polymerization, and acetylation/sulfation levels of fragments obtained by *Hep* I digestion suggest substantial structural diversity within BKHS. These results seem inconsistent with the hypothesis that commercially available BKHS possesses well defined sequences, although this may hold true for chains isolated from a single glycosylation site in specific cells. NS domains obtained after digestion of BKHS with *Hep* III present a maximum length of 12 saccharides, and show variable degrees of sulfation. Overall, the results indicate that in BKHS, NS domains are shorter, while NA and transition domains are longer and of comparable length. Analysis of saturated fragments obtained by *Hep* I or *Hep* III digestion shows that non-reducing ends of BKHS contain NS sequences ranging from 8-mer to 12-mer, or transition domains up to 8-mer long. Although quantitative GPC-MS analysis indicates that non-reducing ends contain *N*-acetylglucosamine in significant amounts, no extended NA domains are detected. The longest NS domain identified at the non-reducing end of BKHS is a dodecasaccharide [47], which is also the maximum length of the NS domains observed within the BKHS chain.

Differently from non-reducing ends, which are constituted by NS or transition domains, reducing ends of BKHS are believed to contain mainly NA domains [48]. In support of this conclusion GPC-MS analysis of BKHS digested with *Hep* III shows fragments belonging to the linkage region, indicating that such residues are most likely immediately preceded by non-sulfated disaccharides. GPC-MS also reveals traces of linkage region fragment where xylose carries a phosphate group. To our knowledge, although phosphorylated linkage region was previously observed in bovine lung HS [39], this is the first report of the presence of phosphate groups in BKHS.

Implementation of orthogonal analytical techniques, such as outlined here, provides a coherent strategy to address HS structure, even in the context of a mixture of chains. Furthermore, we have demonstrated in a separate study (recently published in Scientific Reports [49]) how mathematical modeling can be applied to orthogonal analytical measurements, like those described here, to estimate mixture properties of a complex mixture like heparan sulfate. Combined with structural tools, including array technologies, analysis of HS topology, as well as analysis of HS-protein interactions through crystallography [50] and NMR [51], the ability to elucidate more general structure-activity relationships for this class of molecules should be increased substantially by the application of such an approach.

## Electronic supplementary material

ESM 1 (DOCX 70.6 kb)
