# Supplementary material for: An integrated approach using orthogonal analytical techniques to characterize heparan sulfate structure
Source: Glycoconj J. 2016 Oct 22;34(1):107–17. doi: 10.1007/s10719-016-9734-7 (PMC5266780; doi:10.1007/s10719-016-9734-7)
Supplement: Supplementary file 1 — (DOCX 70.6 kb) [file 10719_2016_9734_MOESM1_ESM.docx]

**Supplementary Information**

**An Integrated Approach Using Orthogonal Analytical Techniques to Characterize Heparan Sulfate Structure**

Daniela Beccati^1,2^, Miroslaw Lech^1,2^, Jennifer Ozug^1^, Nur Sibel Gunay^1^, Jing Wang^1^, Elaine Y Sun^1^, Joel R Pradines^1^, Victor Farutin^1^, Zachary Shriver^1^, Ganesh V Kaundinya^1^, Ishan Capila^1*^

^1^Momenta Pharmaceuticals Inc., 675 West Kendall Street, Cambridge, MA 02142, ^2^ These authors contributed equally to this work

^🞸^To whom correspondence should be addressed. Current address: 675 West Kendall Street, Cambridge, MA 02142, USA. Phone: 1-617-395-5100. Fax: 1-617-621-0431. Email: [icapila@momentapharma.com](mailto:icapila@momentapharma.com)

*GPC-MS Analysis of BKHS Digested with Hep I and Hep III*

Fragments generated by digestion of BKHS with *Hep* I and *Hep* III were analyzed by GPC-MS to determine MW, composition, and relative abundance (**Supplementary Tables 5** and **6**). It is known that the ionization potential of saccharides is strongly dependent on the number of sulfate groups they contain: saccharides with no sulfate groups have very low ionization efficiency while saccharides with a high number of sulfate groups are easily ionized. Experimentally, using isolated saccharides, it was established that differences in ionization due to sulfation play a more significant role in shorter fragments, e.g., in disaccharides. This finding is supported by the fact that the relative ratio between disaccharides as determined by on-line UV and MS shows significant discrepancies (see **Supplementary Tables 3** and **4**). Since UV absorbance is not influenced by the sulfation levels of disaccharides, it constitutes a more reliable method of quantitation. To compensate for the differences observed between the two techniques, correction factors are determined and applied to the ion intensity of saturated and unsaturated disaccharides.

Comparison between UV quantitation and GPC-MS data indicate that the relative amount within the disaccharide group is underestimated for disaccharides with two sulfates, and slightly overestimated for structures with three sulfate groups. Assuming that the correct ratio between GPC-MS intensity and UV absorbance in this experiment corresponding to ionization efficiency representative of true disaccharide abundances in the mixture is the average of these two ratios:

$\mathrm{Average} \left( \frac{MS Intensity Dp2, Ac0, S2, \Delta}{UV Abs Dp2, Ac0, S2, \Delta}, \frac{MS Intensity Dp2, Ac0, S3, \Delta}{UV Abs Dp2, Ac0, S3, \Delta} \right)=$ 1300

where UV Abs and MS Intensity are calculated for the disaccharides obtained by digestion of BKHS with *Hep* I (**Supplementary Tables 3** and **5**, respectively), the following correction factors for GPC-MS derived abundances of disaccharides can be derived.

Based on results obtained by *Hep* I digestion, the correction factors for disaccharides with one sulfate group:

$$1=1300*\frac{UV Abs Dp2,Ac0/1, S1, \Delta}{MS Int Dp2,Ac0/1, S1, \Delta}=2.1$$

with two sulfate groups:

$$2=1300*\frac{UV Abs Dp2,Ac0, S2, \Delta}{MS Int Dp2,Ac0, S2, \Delta}=1.4$$

with three sulfate groups:

$$3=1300*\frac{UV Abs Dp2,Ac0, S3, \Delta}{MS Int Dp2,Ac0, S3, \Delta}=0.8$$

For disaccharides with no sulfate groups, the correction factor is calculated using data from sample digested with *Hep* III (see **Supplementary Table 4** and **6**):

$$0=1300*\frac{UV Abs Dp2,Ac0, S0, \Delta}{MS Int Dp2,Ac0, S0, \Delta}=8.0$$

After application of correction factors to the ion intensity of saturated and unsaturated disaccharides, the relative abundances of each fragment obtained after digestion of BKHS with *Hep* I is determined as reported in **Supplementary Table 5.** Correction factors determined using data from *Hep* I digestion, once applied to GPC-MS abundances in *Hep* III digest, improve agreement with UV data **(Supplementary Table** **6)**.

***Supplementary Results***

**Supplementary Table 1**: Monosaccharide composition of BKHS calculated by IP-RPHPLC and 2D NMR (HSQC) data integration

| **Monosaccharides** | **IP-RPHPLC** | **2D NMR** |
| --- | --- | --- |
|  | Glucosamine Mol % | |
| H_NS_ | 35.4 | 31.7 |
| H_NAc_ | 63.9 | 66.2 |
| H_NS3S_ | 0.7 | 0.6 |
|  | 6-*O*-Sulfation % | |
| 6-*O*-Sulfation | 22.2 | 25.0 |
|  | Uronic Acid Mol % | |
| I_2S_ | 13.5 | 17.8 |
| I/G | 86.0 | 81.5 |
| Epoxide | N/A^1^ | 0.7 |
| GalA | 0.6^1^ | n.d. |

^1^ Heparin lyase enzymes convert epoxide into galacturonic acid

**Supplementary Table 2**: LC-MS analysis of BKHS digested with heparin lyase enzymes

| **Identity** | **Calculated MW** |
| --- | --- |
| **Monosaccharides** |  |
| H_NS6S_ or H_NS3S_ | 338.99 |
| H_NAc6S_ | 301.05 |
| **Unsaturated disaccharides** |  |
| ΔU-H_NH2_ | 337.10 |
| ΔU-H_NAc_ | 379.11 |
| ΔU_gal_-H_NS_ | 417.06 |
| ΔU-H_NAc6S_ | 459.07 |
| ΔU_2S_-H_NAc_ | 459.07 |
| ΔU-H_NS_ | 417.06 |
| ΔU-H_NS6S_ | 497.01 |
| ΔU_2S_-H_NS_ | 497.01 |
| ΔU-H_NS3S_ | 497.01 |
| ΔU_2S_-H_NAc6S_ | 539.03 |
| ΔU_2S_-H_NS6S_ | 576.97 |
| Dp2, 0Ac, S3, Δ | 576.97 |
| ΔU_2S_-H_NS6S3S_ | 656.93 |
| **Unsaturated trisaccharides** |  |
| ΔU-H_NAc_-G | 555.14 |
| Dp3, 0Ac, S3, Δ | 753.00 |
| **Unsaturated tetrasaccharides** |  |
| Dp4, 0Ac, S2, Δ | 834.12 |
| Dp4, 0Ac, S2, Δ, -18Da^1^ | 816.10 |
| Dp4, 1Ac, S2, Δ | 876.13 |
| Dp4, 0Ac, S3, Δ, -18Da^1^ | 896.06 |
| Dp4, 0Ac, S3, Δ | 914.07 |
| ΔU-H_NAc6S_-Glc-H_NS3S_ | 956.08 |
| ΔU-H_NAc6S_-Glc-H_NS3S6S_ | 1036.04 |
| Dp4, 0Ac, S4, Δ , -18Da^1^ | 976.02 |
| Dp4, 0Ac, S4, Δ | 994.03 |
| Dp4, 0Ac, S5, Δ | 1073.99 |
| Dp4, 0Ac, S5, Δ, -18Da^1^ | 1055.98 |
| **Saturated saccharides starting with glucosamine (H-U-)** |  |
| Dp3, 0Ac, S2 | 676.09 |
| Dp3, 0Ac, S3 | 756.05 |
| Dp3, 1Ac, S3 | 798.06 |
| Dp3, 1Ac, S4 | 878.02 |
| Dp3, 0Ac, S4 | 836.01 |
| Dp3, 0Ac, S5 | 915.96 |
| **Saturated saccharides starting with uronic acid (U-H-)** |  |
| Dp2, 1Ac, S0 | 397.12 |
| Dp2, 0Ac, S1 | 435.07 |
| Dp2, 1Ac, S1 | 477.08 |
| Dp2, 0Ac, S2 | 515.03 |
| Dp2, 0Ac, S3 | 594.98 |
| Dp4, 1Ac, S4 | 1054.05 |
| Dp4, 0Ac, S3 | 932.08 |
| Dp4, 1Ac, S3 | 974.09 |
| Dp4, 0Ac, S4 | 1012.04 |
| Dp4, 0Ac, S5 | 1092.00 |
| **Linkage region** |  |
| ΔU-Gal-Gal-Xyl-OH | 632.18 |

^1^ -18Da: could indicate epoxide or loss of water

**Supplementary Figure 1**: GPC profile (monitored at 232 nm) of BKHS digested with *Hep* I. Regions integrated to calculate chain distributions are indicated.


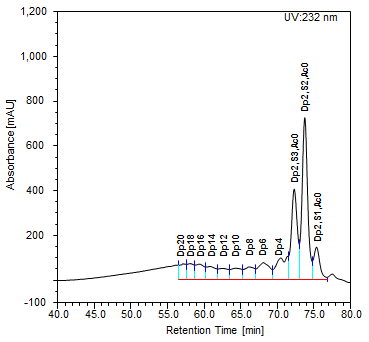


**Supplementary Table 3**: Fragment distribution of BKHS digested by *Hep* I

| **Composition** | **Area mAU*min** | **Rel. Area %** |
| --- | --- | --- |
| Dp20 | 75.69 | 3.6 |
| Dp18 | 80.53 | 3.9 |
| Dp16 | 95.34 | 4.6 |
| Dp14 | 88.53 | 4.2 |
| Dp12 | 76.96 | 3.7 |
| Dp10 | 88.00 | 4.2 |
| Dp8 | 90.53 | 4.3 |
| Dp6 | 136.62 | 6.6 |
| Dp4 | 178.37 | 8.6 |
| Dp2,S3,Ac0^1^ | 380.23 | 18.2 |
| Dp2,S2,Ac0^1^ | 649.69 | 31.2 |
| Dp2,S1,Ac0/1^1,2^ | 143.61 | 6.9 |

^1^ The number of sulfates and acetyl groups is determined by MS analysis

^2^ Dp2,S1,Ac0 = ΔUH_NS_; Dp2,S1,1Ac = ΔU_2S_H_NAc_ or ΔUH_NAc6S_. These residues indicate that *Hep* I does not possess exclusive specificity for sulfated residues.

**Supplementary Figure 2**: GPC profile (monitored at 232 nm) of BKHS digested with *Hep* III. Regions integrated to calculate chain distributions are indicated.


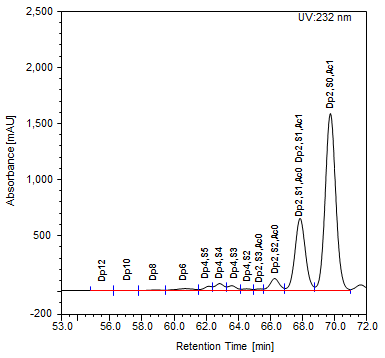


**Supplementary Table 4**: Fragment distribution of BKHS digested with *Hep* III

| **Composition** | **Area mAU*min** | **Rel. Area %** |
| --- | --- | --- |
| Dp12 | 0.69 | < 0.1 |
| Dp10 | 3.55 | 0.2 |
| Dp8 | 11.22 | 0.6 |
| Dp6 | 32.02 | 1.6 |
| Dp4,S5 | 23.74 | 1.2 |
| Dp4,S4 | 41.93 | 2.2 |
| Dp4,S3 | 28.72 | 1.5 |
| Dp4,S2 | 11.90 | 0.6 |
| Dp2,S3,Ac0^1,2^ | 10.22 | 0.5 |
| Dp2,S2,Ac0^1^ | 78.30 | 4.0 |
| Dp2,S1,Ac0/1^1^ | 492.55 | 25.3 |
| Dp2,S0,Ac1^1^ | 1213.63 | 62.3 |

^1^ The number of sulfates and acetyl groups is determined by MS analysis

^2^ Dp2,S3,Ac0 = ΔU_2S_H_NS6S_. This residue indicates that *Hep* I does not possess complete specificity for non-sulfated residues.

**Supplementary Table 5**: Chain distribution by GPC-MS of BKHS after *Hep* I digestion

| **Identity** | **MW** | **MS Intensity^&^** | **Relative %** |
| --- | --- | --- | --- |
| H, Dp1, Ac0, S2 | 339.00 | 39013 | 1.19 |
| Dp2, Ac0, S1, Δ | 417.10 | 44905 (* 2.1) | 2.89 |
| Dp2, Ac0, S1, saturated | 435.07 | 17706 (* 2.1) | 1.14 |
| Dp2, Ac1, S1, Δ | 459.07 | 44774 (*2.1) | 2.88 |
| Dp2, Ac0, S2, saturated | 515.08 | 57774 (*1.4) | 2.48 |
| Dp2, Ac0, S2, Δ | 497.01 | 620512 (*1.4) | 26.61 |
| Dp2, Ac0, S3, Δ | 576.98 | 643272 (*0.8) | 15.76 |
| ΔU_2S_-H_NS6S3S_ | 656.93 | 51017 | 1.56 |
| Dp3, Ac0, S2, H-U-H | 676.07 | 2786 | 0.09 |
| Dp3, Ac0, S3, H-U-H | 756.01 | 23237 | 0.71 |
| Dp3, Ac0, S4, H-U-H | 836.00 | 12796 | 0.39 |
| Dp3, Ac1, S2, H-U-H | 718.09 | 50746 | 1.55 |
| Dp3, Ac1, S3, H-U-H | 798.04 | 33496 | 1.03 |
| Dp3, Ac1, S4, H-U-H | 878.01 | 3802 | 0.12 |
| Dp4, Ac1, S2, Δ | 876.11 | 82089 | 2.51 |
| Dp4, Ac1, S2, saturated | 894.13 | 30831 | 0.94 |
| Dp4, Ac0, S3, Δ, -18 Da^1^ | 896.05 | 40105 | 1.23 |
| Dp4, Ac0, S3, Δ | 914.05 | 68413 | 2.10 |
| Dp4, Ac0, S3, saturated | 932.07 | 13655 | 0.42 |
| Dp4, Ac1, S3, Δ | 956.09 | 88987 | 2.73 |
| Dp4, Ac1, S3, saturated | 974.07 | 12402 | 0.38 |
| Dp4, Ac1, S4, Δ | 1036.00 | 5960 | 0.18 |
| Dp4, Ac0, S4, Δ, -18 Da^1^ | 976.01 | 13064 | 0.40 |
| Dp4, Ac0, S4, Δ | 994.01 | 21328 | 0.65 |
| Dp4, Ac0, S4, saturated | 1012.00 | 5709 | 0.17 |
| Dp4, Ac0, S5, Δ, -18 Da^1^ | 1055.92 | 3589 | 0.11 |
| Dp4, Ac0, S5, Δ | 1073.95 | 6251 | 0.19 |
| Dp5, Ac1,S3, H-U-H-U-H | 1135.15 | 7985 | 0.24 |
| Dp5, Ac0,S4, H-U-H-U-H | 1173.07 | 6640 | 0.20 |
| Dp5, Ac2,S3, H-U-H-U-H | 1177.12 | 4072 | 0.12 |
| Dp6, Ac2, S1, Δ* | 1175.27 | 7836 | 0.24 |
| Dp6, Ac1, S2, Δ | 1213.21 | 4612 | 0.14 |
| Dp6, Ac1, S3, Δ | 1293.15 | 58027 | 1.78 |
| Dp6, Ac1, S3, saturated | 1311.16 | 4390 | 0.13 |
| Dp6, Ac1, S4, Δ | 1373.13 | 80577 | 2.47 |
| Dp6, Ac1, S4, saturated | 1391.11 | 2797 | 0.09 |
| Dp6, Ac0, S4, Δ, -2x18 Da^1^ | 1295.02 | 3354 | 0.10 |
| Dp6, Ac0, S4, Δ, -18 Da^1^ | 1313.08 | 6567 | 0.20 |
| Dp6, Ac0, S4, Δ | 1331.09 | 12396 | 0.38 |
| Dp6, Ac1, S5, Δ | 1453.06 | 39262 | 1.20 |
| Dp6, Ac2, S2, Δ* | 1255.21 | 33137 | 1.01 |
| Dp6, Ac2, S2, saturated* | 1273.23 | 3414 | 0.10 |
| Dp6, Ac2, S3, Δ* | 1335.17 | 29272 | 0.90 |
| Dp6, Ac2, S4, Δ* | 1415.11 | 8594 | 0.26 |
| Dp8, Ac1, S4, Δ | 1710.19 | 8323 | 0.25 |
| Dp8, Ac1, S5, Δ | 1790.17 | 12346 | 0.38 |
| Dp8, Ac1, S6, Δ | 1870.06 | 3758 | 0.12 |
| Dp8, Ac2, S2, Δ | 1592.32 | 6565 | 0.20 |
| Dp8, Ac2, S3, Δ | 1672.24 | 40015 | 1.23 |
| Dp8, Ac2, S4, Δ | 1752.22 | 55393 | 1.70 |
| Dp8, Ac2, S4, saturated | 1770.22 | 756 | 0.02 |
| Dp8, Ac2, S5, Δ | 1832.20 | 18296 | 0.56 |
| Dp8, Ac2, S6, Δ | 1912.11 | 3741 | 0.11 |
| Dp8, Ac3, S2, Δ* | 1634.32 | 17072 | 0.52 |
| Dp8, Ac3, S3, Δ* | 1714.27 | 25190 | 0.77 |
| Dp8, Ac3, S4, Δ* | 1794.19 | 7826 | 0.24 |
| Dp10, Ac2, S3, Δ | 2009.38 | 2441 | 0.07 |
| Dp10, Ac2, S4, Δ | 2089.33 | 10543 | 0.32 |
| Dp10, Ac2, S5, Δ | 2169.28 | 14087 | 0.43 |
| Dp10, Ac2, S6, Δ | 2249.21 | 15518 | 0.48 |
| Dp10, Ac2, S7, Δ | 2329.16 | 3320 | 0.10 |
| Dp10, Ac3, S2, Δ | 1971.43 | 4424 | 0.14 |
| Dp10, Ac3, S3, Δ | 2051.38 | 25442 | 0.78 |
| Dp10, Ac3, S4, Δ | 2131.33 | 30791 | 0.94 |
| Dp10, Ac3, S5, Δ | 2211.28 | 18923 | 0.58 |
| Dp10, Ac3, S6, Δ | 2291.29 | 2221 | 0.07 |
| Dp10, Ac4, S2, Δ* | 2013.43 | 12106 | 0.37 |
| Dp10, Ac4, S3, Δ* | 2093.38 | 14372 | 0.44 |
| Dp10, Ac4, S4, Δ* | 2173.33 | 4151 | 0.13 |
| Dp12, Ac2, S5, Δ | 2506.37 | 1889 | 0.06 |
| Dp12, Ac2, S6, Δ | 2586.29 | 2450 | 0.08 |
| Dp12, Ac2, S7, Δ | 2666.25 | 1176 | 0.04 |
| Dp12, Ac3, S4, Δ | 2468.41 | 8343 | 0.26 |
| Dp12, Ac3, S5, Δ | 2548.37 | 14106 | 0.43 |
| Dp12, Ac3, S6, Δ | 2628.33 | 10297 | 0.32 |
| Dp12, Ac3, S7, Δ | 2708.25 | 2563 | 0.08 |
| Dp12, Ac4, S3, Δ | 2430.45 | 12790 | 0.39 |
| Dp12, Ac4, S4, Δ | 2510.41 | 18073 | 0.55 |
| Dp12, Ac4, S5, Δ | 2590.37 | 9465 | 0.29 |
| Dp12, Ac4, S6, Δ | 2670.33 | 2158 | 0.07 |
| Dp14, Ac3, S5, Δ | 2885.41 | 1543 | 0.05 |
| Dp14, Ac3, S6, Δ | 2965.37 | 3189 | 0.10 |
| Dp14, Ac3, S7, Δ | 3045.31 | 2012 | 0.06 |
| Dp14, Ac4, S4, Δ | 2847.49 | 7501 | 0.23 |
| Dp14, Ac4, S5, Δ | 2927.45 | 11850 | 0.36 |
| Dp14, Ac4, S6, Δ | 3007.45 | 8928 | 0.27 |
| Dp14, Ac4, S7, Δ | 3087.31 | 2550 | 0.08 |
| Dp14, Ac5, S3, Δ | 2809.61 | 6706 | 0.21 |
| Dp14, Ac5, S4, Δ | 2889.53 | 12028 | 0.37 |
| Dp14, Ac5, S5, Δ | 2969.49 | 8815 | 0.27 |
| Dp14, Ac5, S6, Δ | 3049.36 | 2709 | 0.08 |
| Dp16, Ac4, S4, Δ | 3184.61 | 710 | 0.02 |
| Dp16, Ac4, S5, Δ | 3264.57 | 2027 | 0.06 |
| Dp16, Ac4, S6, Δ | 3344.53 | 3113 | 0.10 |
| Dp16, Ac4, S7, Δ | 3424.51 | 3092 | 0.09 |
| Dp16, Ac4, S8, Δ | 3504.49 | 1402 | 0.04 |
| Dp16, Ac5, S3, Δ | 3146.73 | 957 | 0.03 |
| Dp16, Ac5, S4, Δ | 3226.65 | 5015 | 0.15 |
| Dp16, Ac5, S5, Δ | 3306.61 | 7082 | 0.22 |
| Dp16, Ac5, S6, Δ | 3386.57 | 6383 | 0.20 |
| Dp16, Ac5, S7, Δ | 3466.51 | 3598 | 0.11 |
| Dp16, Ac6, S3, Δ | 3188.69 | 6448 | 0.20 |
| Dp16, Ac6, S4, Δ | 3268.65 | 6475 | 0.20 |
| Dp16, Ac6, S5, Δ | 3348.57 | 6925 | 0.21 |
| Dp16, Ac7, S2, Δ* | 3150.73 | 5447 | 0.17 |
| Dp16, Ac7, S3, Δ* | 3230.71 | 3655 | 0.11 |
| Dp18, Ac5, S6, Δ | 3723.61 | 2757 | 0.08 |
| Dp18, Ac5, S7, Δ | 3803.56 | 2445 | 0.07 |
| Dp18, Ac5, S8, Δ | 3883.51 | 1589 | 0.05 |
| Dp18, Ac6, S4, Δ | 3605.77 | 3029 | 0.09 |
| Dp18, Ac6, S5, Δ | 3685.71 | 3919 | 0.12 |
| Dp18, Ac6, S6, Δ | 3765.66 | 5106 | 0.16 |
| Dp18, Ac6, S7, Δ | 3845.56 | 1797 | 0.06 |
| Dp18, Ac7, S3, Δ | 3567.77 | 5229 | 0.16 |
| Dp18, Ac7, S4, Δ | 3647.73 | 4311 | 0.13 |
| Dp18, Ac7, S5, Δ | 3727.66 | 3347 | 0.10 |
| Dp18, Ac7, S6, Δ | 3807.61 | 1185 | 0.04 |
| Dp18, Ac8, S2, Δ* | 3529.81 | 4440 | 0.14 |
| Dp18, Ac8, S3, Δ* | 3609.77 | 3829 | 0.12 |
| Dp20, Ac5, S7, Δ | 4140.71 | 1356 | 0.04 |
| Dp20, Ac5, S8, Δ | 4220.59 | 794 | 0.02 |
| Dp20, Ac6, S5, Δ | 4022.76 | 1854 | 0.06 |
| Dp20, Ac6, S6, Δ | 4102.81 | 3041 | 0.09 |
| Dp20, Ac6, S7, Δ | 4182.61 | 2169 | 0.07 |
| Dp20, Ac6, S8, Δ | 4262.59 | 2074 | 0.06 |
| Dp20, Ac7, S4, Δ | 3984.85 | 2666 | 0.08 |
| Dp20, Ac7, S5, Δ | 4064.66 | 4676 | 0.14 |
| Dp20, Ac7, S6, Δ | 4144.69 | 3706 | 0.11 |
| Dp20, Ac7, S7, Δ | 4224.61 | 1915 | 0.06 |
| Dp20, Ac7, S8, Δ | 4304.47 | 718 | 0.02 |
| Dp20, Ac8, S3, Δ | 3946.89 | 3663 | 0.11 |
| Dp20, Ac8, S4, Δ | 4026.81 | 4014 | 0.12 |
| Dp20, Ac9, S2, Δ* | 3908.97 | 3033 | 0.09 |
| Dp20, Ac9, S3, Δ* | 3988.93 | 3126 | 0.10 |

^&^ Values in brackets report the correction factors applied to calculate relative percentages

^1^ -18Da: could indicate presence of epoxide or loss of water

* NA domains (see text for explanation)

**Supplementary Table 6**: Chain distribution by GPC-MS of BKHS after *Hep* III digestion

| **Identity** | **MW** | **MS Intensity^&^** | **Relative %** |
| --- | --- | --- | --- |
| H, Dp1, Ac1, S1 | 301.03 | 1896 | 0.07 |
| Dp2, Ac1, S0, Δ | 379.10 | 196957 (*8.0) | 54.45 |
| Dp2, Ac1, S0, saturated | 397.11 | 3003 (*8.0) | 0.83 |
| Dp2, Ac0, S1, Δ | 417.05 | 142630 (*2.1) | 10.35 |
| Dp2, Ac1, S1, Δ | 459.07 | 147290 (*2.1) | 10.69 |
| Dp2, Ac0, S2, Δ | 497.02 | 88305 (*1.4) | 4.27 |
| Dp2, Ac0, S2, saturated | 514.98 | 5186 (*1.4) | 0.25 |
| Dp2, Ac0, S3, Δ | 576.94 | 1496 (*0.8) | 0.04 |
| Dp3, Ac0, S2, H-U-H | 676.06 | P | P |
| Dp3, Ac0, S3, H-U-H | 756.04 | 840 | 0.03 |
| Dp3, Ac0, S4, H-U-H | 836.00 | 763 | 0.03 |
| Dp3, Ac0, S5, H-U-H | 915.96 | 719 | 0.02 |
| ΔU-Gal-Gal-Xyl-OH | 632.21 | 3631 | 0.13 |
| ΔU-Gal-Gal-Xyl-OH +80Da | 712.14 | P | P |
| Dp4, Ac1, S2, Δ | 876.12 | 31797 | 1.10 |
| Dp4, Ac0, S3, -18Da^1^ | 896.06 | 8780 | 0.30 |
| Dp4, Ac0, S3, Δ | 914.01 | 75477 | 2.61 |
| Dp4, Ac1, S3, Δ | 956.08 | 4581 | 0.16 |
| Dp4, Ac0, S4, Δ | 994.04 | 109702 | 3.79 |
| Dp4, Ac0, S5, -18Da^1^ | 1055.94 | 127 | < 0.01 |
| Dp4, Ac0, S5, Δ | 1073.94 | 72160 | 2.49 |
| Dp4, Ac0, S6, Δ | 1153.91 | 6366 | 0.22 |
| Dp6, Ac0, S4, -18Da^1^ | 1313.13 | 4873 | 0.17 |
| Dp6, Ac0, S4, Δ | 1331.13 | 3784 | 0.13 |
| Dp6, Ac1, S4, Δ | 1373.16 | 2055 | 0.07 |
| Dp6, Ac0, S5, -18Da^1^ | 1393.08 | 5243 | 0.18 |
| Dp6, Ac0, S5, Δ | 1411.09 | 27608 | 0.95 |
| Dp6, Ac1, S5, Δ | 1453.11 | 3204 | 0.11 |
| Dp6, Ac0, S6, Δ | 1491.00 | 37407 | 1.29 |
| Dp6, Ac0, S7, Δ | 1570.96 | 29389 | 1.02 |
| Dp6, Ac0, S8, Δ | 1650.92 | 16383 | 0.57 |
| Dp8, Ac0, S6, -18Da^1^ | 1810.15 | 3188 | 0.11 |
| Dp8, Ac0, S6, Δ | 1828.14 | 3948 | 0.14 |
| Dp8, Ac2, S6, saturated | 1930.15 | 3044 | 0.11 |
| Dp8, Ac0, S7, -18Da^1^ | 1890.11 | 3914 | 0.14 |
| Dp8, Ac0, S7, Δ | 1908.11 | 15213 | 0.53 |
| Dp8, Ac0, S7, saturated | 1926.12 | 4012 | 0.14 |
| Dp8, Ac1, S7, Δ | 1950.12 | 1682 | 0.06 |
| Dp8, Ac0, S8, -18Da^1^ | 1970.07 | 2725 | 0.09 |
| Dp8, Ac0, S8, Δ | 1988.00 | 16501 | 0.57 |
| Dp8, Ac0, S8, saturated | 2006.01 | 5941 | 0.21 |
| Dp8, Ac0, S9, Δ | 2068.02 | 10131 | 0.35 |
| Dp8, Ac0, S9, saturated | 2086.00 | 3468 | 0.12 |
| Dp8, Ac0, S10, Δ | 2147.93 | 4678 | 0.16 |
| Dp8, Ac0, S10, saturated | 2166.04 | 2032 | 0.07 |
| Dp10, Ac0, S8, -18Da^1^ | 2307.14 | 1651 | 0.06 |
| Dp10, Ac0, S8, Δ | 2325.14 | 1437 | 0.05 |
| Dp10, Ac0, S9, -18Da^1^ | 2387.14 | 1721 | 0.06 |
| Dp10, Ac0, S9, Δ | 2405.10 | 5089 | 0.18 |
| Dp10, Ac0, S9, saturated | 2423.11 | 1985 | 0.07 |
| Dp10, Ac0, S10, -18Da^1^ | 2467.14 | 672 | 0.02 |
| Dp10, Ac0, S10, Δ | 2485.06 | 4474 | 0.15 |
| Dp10, Ac0, S10, saturated | 2503.10 | 2469 | 0.09 |
| Dp10, Ac0, S11, Δ | 2565.05 | 2136 | 0.07 |
| Dp10, Ac0, S11, saturated | 2583.05 | 1132 | 0.04 |
| Dp10, Ac0, S12, Δ | 2645.04 | 720 | 0.02 |
| Dp12, Ac0, S11, -18Da^1^ | 2884.19 | 305 | 0.01 |
| Dp12, Ac0, S11, Δ | 2902.14 | 711 | 0.02 |
| Dp12, Ac0, S11, saturated | 2920.05 | 466 | 0.02 |
| Dp12, Ac0, S12, Δ | 2982.12 | 687 | 0.02 |
| Dp12, Ac0, S12, saturated | 3000.06 | 492 | 0.02 |

^&^ Values in brackets report the correction factors that are applied to calculate relative percentages

^1^ -18Da: could indicate presence of epoxide or loss of water

P: present but below the limit of quantitation
